# Contrast-enhanced ultrasound evaluation of pancreatic cancer xenografts in nude mice after irradiation with sub-threshold focused ultrasound for tumor ablation

**DOI:** 10.18632/oncotarget.16621

**Published:** 2017-03-28

**Authors:** Yi Hui Gao, Lei Wu, Rui Wang, Qian Guo, Yi Ni Chen, Bing Hu, Li Xin Jiang

**Affiliations:** ^1^ Department of Ultrasonography, Shanghai Jiaotong University Affiliated No. 6 Hospital, Shanghai 200233, PR China; ^2^ Shanghai Institute of Ultrasound in Medicine, Shanghai 200233, PR China

**Keywords:** dynamic contrast-enhanced ultrasound, pancreatic cancer, sub-threshold focused ultrasound, evaluation with DCE-US, B-mode US examination

## Abstract

We evaluated the efficacy of contrast-enhanced ultrasound for assessing tumors after irradiation with sub-threshold focused ultrasound (FUS) ablation in pancreatic cancer xenografts in nude mice. Thirty tumor-bearing nude mice were divided into three groups: Group A received sham irradiation, Group B received a moderate-acoustic energy dose (sub-threshold), and Group C received a high-acoustic energy dose. In Group B, B-mode ultrasound (US), color Doppler US, and dynamic contrast-enhanced ultrasound (DCE-US) studies were conducted before and after irradiation. After irradiation, tumor growth was inhibited in Group B, and the tumors shrank in Group C. In Group A, the tumor sizes were unchanged. In Group B, contrast-enhanced ultrasound (CEUS) images showed a rapid rush of contrast agent into and out of tumors before irradiation. After irradiation, CEUS revealed contrast agent perfusion only at the tumor periphery and irregular, un-perfused volumes of contrast agent within the tumors. DCE-US perfusion parameters, including peak intensity (PI) and area under the curve (AUC), had decreased 24 hours after irradiation. PI and AUC were increased 48 hours and 2weeks after irradiation. Time to peak (TP) and sharpness were increased 24 hours after irradiation. TP decreased at 48 hours and 2 weeks after irradiation. CEUS is thus an effective method for early evaluation after irradiation with sub-threshold FUS.

## INTRODUCTION

High-intensity focused ultrasound (HIFU) irradiation is a non-invasive tumor treatment method. Currently, the main clinical application of HIFU is the treatment of benign and malignant solid tumors, such as uterine fibroids, pancreatic cancer, liver cancer, and osteosarcoma [1]. The pancreas is a deeply located, retroperitoneal organ that is surrounded by the gastrointestinal tract, blood vessels, and the biliary tract. Animal experiments and clinical studies have confirmed that during HIFU irradiation, an excessive acoustic energy dose can cause serious side effects, such as damage to normal tissues outside of the target area [2, 3]. Reducing the acoustic energy dose of HIFU can prevent adverse reactions but can cause tissue damage by inducing apoptosis [4].

Until now, no appropriate methods to detect tumor cell apoptosis, necrosis, and cellular function changes in the early phase after HIFU irradiation (within 1 week) were available [5, 6]. Because of the edema and inflammatory cell infiltration of tissues surrounding the ablation lesions during the early phase after HIFU, B-mode ultrasound (US) and color Doppler US cannot accurately determine the extent of ablation lesions and irradiation effects. Furthermore, some studies have shown that increased tissue damage after local hyperthermia is associated with the progression of microvascular injury [7, 8]. Because of the damaging effects of HIFU on the microvasculature of the target tissue, the lesion area might not be limited to the focal point in living tissue [9, 10]. Currently, clinical HIFU irradiation is guided by either magnetic resonance imaging (MRI) or US imaging. MRI-guided focused ultrasound can provide high-resolution images for treatment targeting, temperature monitoring, and post-treatment visualization of the thermal lesion [11]. Despite its advantages, MRI is costly, lacks portability, and has complex electromagnetic compatibility issues. B-mode ultrasound has been another imaging technique commonly used in clinical practice for HIFU monitoring. It has permitted visualization of severe tumors for treatment targeting of tumor sizes and ablation lesions [12].

The recent development of dynamic contrast-enhanced ultrasound (DCE-US) has permitted noninvasive evaluation of tumor perfusion. Its potential use for assessing tumor perfusion has received clinical attention for cancer imaging because it visualizes early changes in tumor perfusion during chemotherapy. Animal studies have shown a reduction in tumor vascularization detected by DCE-US after days or weeks of treatment [13]. DCE-US has also been reported to be a useful diagnostic tool for revealing the vascularity of pancreatic ductal carcinoma. Kim et al [14] reported that DCE-US can assess the early response after combined gemcitabine and low-power HIFU irradiation for the mouse xenograft model of human pancreatic cancer.

We evaluated the efficacy of DCE-US in assessing lesions after irradiation of pancreatic cancer xenografts in nude mice with sub-threshold focused ultrasound (FUS), as well as the role of DCE-US for predicting the early treatment response compared with the pathology results.

## RESULTS

### Temperature measurement and ultrasound acoustic energy dose

Temperatures measured at the foci of the four radiation acoustic energy doses were recorded. The ultrasound radiation acoustic energy dose (T_max_) and temperature change (ΔT) above baseline are shown in Table 1. Pairwise comparisons showed that differences between temperatures of any two acoustic energy doses were statistically significant. (*P* <0.05).

**Table 1:** Parameters of different ultrasound radiation acoustic energy doses and temperatures of different radiation acoustic energy doses measured at the focus

Group	T ON (ms)	T OFF (ms)	Acoustic intensity (w/cm^2^)	T_max_ (°C)	ΔT (°C)
A	500	2000	0	0	0
B	500	2000	1134	57.5 ± 2.2	20.2 ± 1.8
C	500	2000	2835	72.2 ± 4.8	45.5 ± 3.6

T ON is turn-on time and T OFF is turn-off time. T_max_ is the maximum temperature during irradiation, and temperature change (Δ T) is the final temperature rise of cell suspension measured after ultrasound irradiation.

### Skin burn and tumor size after HIFU irradiation

**Figure 1:**
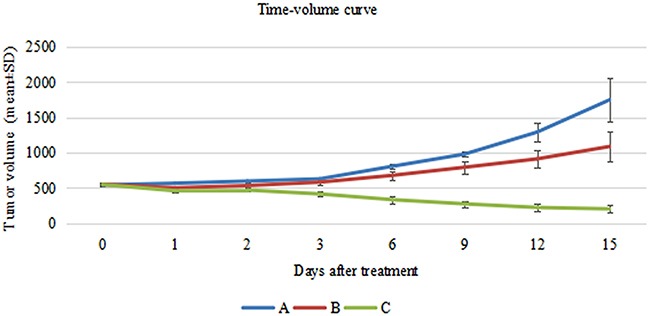
Comparison of change in tumor size between before and after irradiation (mm^3^, mean ± SD) Blue line shows the tumor size without HIFU irradiation in Group **(A)** Red line reflects the trend of tumor volume after sub-threshold HIFU irradiation in Group **(B)** Green line shows the tumor volume in Group **(C)**.

The tumor size for the three groups are shown in Table 2. Before irradiation, the tumor volume was 538.98 ± 15.04 mm^3^ in Group A, 548.72 ± 27.94 mm^3^ in Group B (55°-60° C), and 545.56 ± 22.79 mm^3^ in Group C (65°-80° C). There was no statistical difference in the tumor size among the three groups. After irradiation, the tumor growth was inhibited in Group B and the tumors shrank in Group C, with three tumors disappearing in Group C. Statistically different tumor sizes were seen among the three groups after HIFU irradiation. The tumor sizes of Group B and Group C were smaller than those of the control group. (Figure 1) No damage occurred to normal organs around the tumors in Group B. Three mice had limbs damaged by HIFU irradiation in Group C.

**Table 2:** Comparison of change in tumor size before and after treatment (mm^3^, mean ± SD)

Days after HIFU irradiation								
Group	0	1	2	3	6	9	12	15
A	538.97±15.03	563.97±14.25	593.97±21.25	625.44±28.11	803.84±40.20	977.11±39.57	1288.96±132.30	1746.57±312.76
B	548.72±17.94	499.35±15.94	530.72±27.94	579.25±35.71	675.10±60.9	790.54±84.44	909.28±127.26	1085.23±217.13
C	545.56±18.79	456.72±16.74	467.7±20.94	412.25±30.71	331.10±51.9	271.54±42.44	220.28±57.26	200.23±54.13

Note: Values are expressed as mean ± SD.

### Ultrasound examination follow-up results

**Figure 2:**
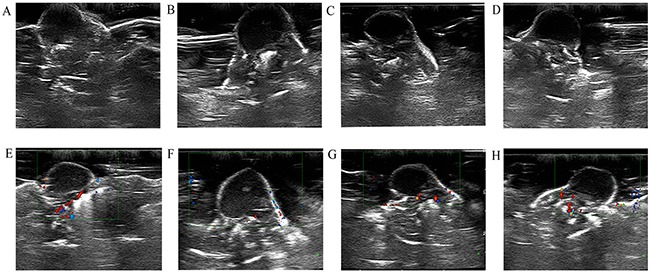
B-mode US and color Doppler flow imaging obtained before HIFU irradiation (**A** and **E**) B-mode US image obtained at 24 hours, 48 hours, and 2 weeks after HIFU irradiation (**B, C, and D**). Color Doppler flow imaging obtained at 24 hours, 48 hours, and 2 weeks after HIFU irradiation (**F, G, and H**).

**Figure 3:**
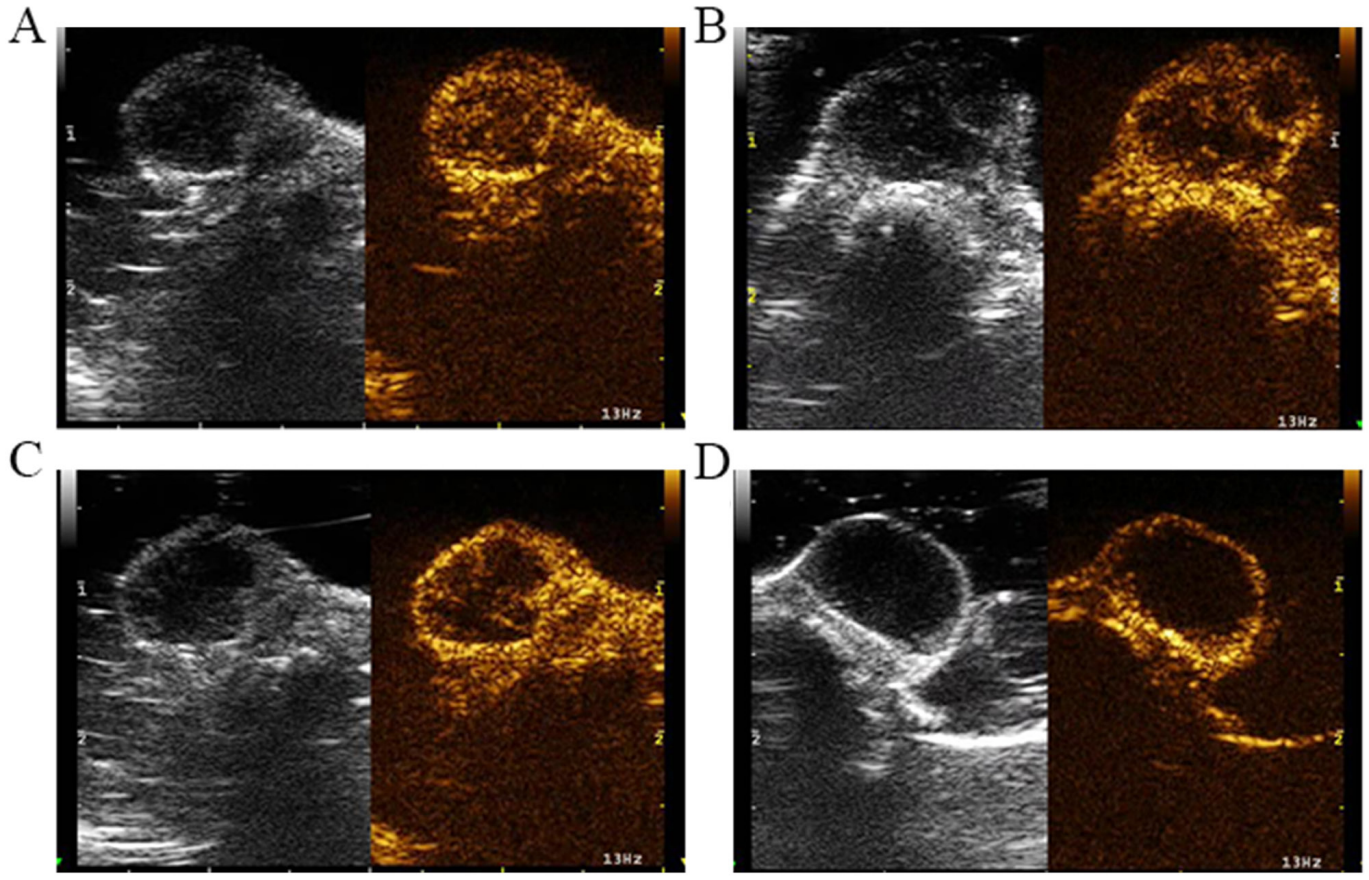
Serial CEUS images before and after irradiation **(A)** CEUS images before HIFU irradiation. CEUS revealed contrast agent perfusion at the periphery of the tumors, as well as irregular contrast agent non-perfused areas within the tumors. **(B)** CEUS images at 24 hours after HIFU irradiation. CEUS revealed contrast agent perfusion at the periphery of the tumors. **(C)** CEUS images at 48 hours after HIFU irradiation. The CEUS results were like those of the 24-hours group, with irregular contrast agent non-perfused areas within the tumors and contrast agent perfusion in the tumor periphery. **(D)** CEUS images at 2 weeks after HIFU irradiation. CEUS revealed the peripheral contrast agent perfusion area was increased compared with the 24-hours and 48-hours groups.

Before irradiation, the skin surface above the subcutaneous pancreatic cancer xenografts in nude mice was undamaged. After irradiation, no mice had skin burns in Group A or Group B, but seven mice in Group C had skin burns. Before irradiation, the skin over the xenografts was hyperechoic. The interior part of the xenografts exhibited low or very low echo. The internal echo of the tumors was homogeneous.(Figure 2A). Color Doppler flow imaging showed that the pancreatic cancer xenografts had no significant internal flow. The flow signals of the tumor-feeding vessels were detected in the periphery of xenografts, and arterial and venous spectra were detected (Figure 2E). CEUS images revealed that at approximately 4 to 6 seconds after bolus injection of the contrast agent, the interior portion of the xenografts showed perfusion with heterogeneous enhancement (Figure 3A). The degree of enhancement was higher than that of the surrounding muscles. At 12 to 15 seconds after injection, the contrast agent inside the tumors began to be expurgated, showing the typical malignant tumor enhancement mode of rapid entry and rapid exit.

**Figure 4:**
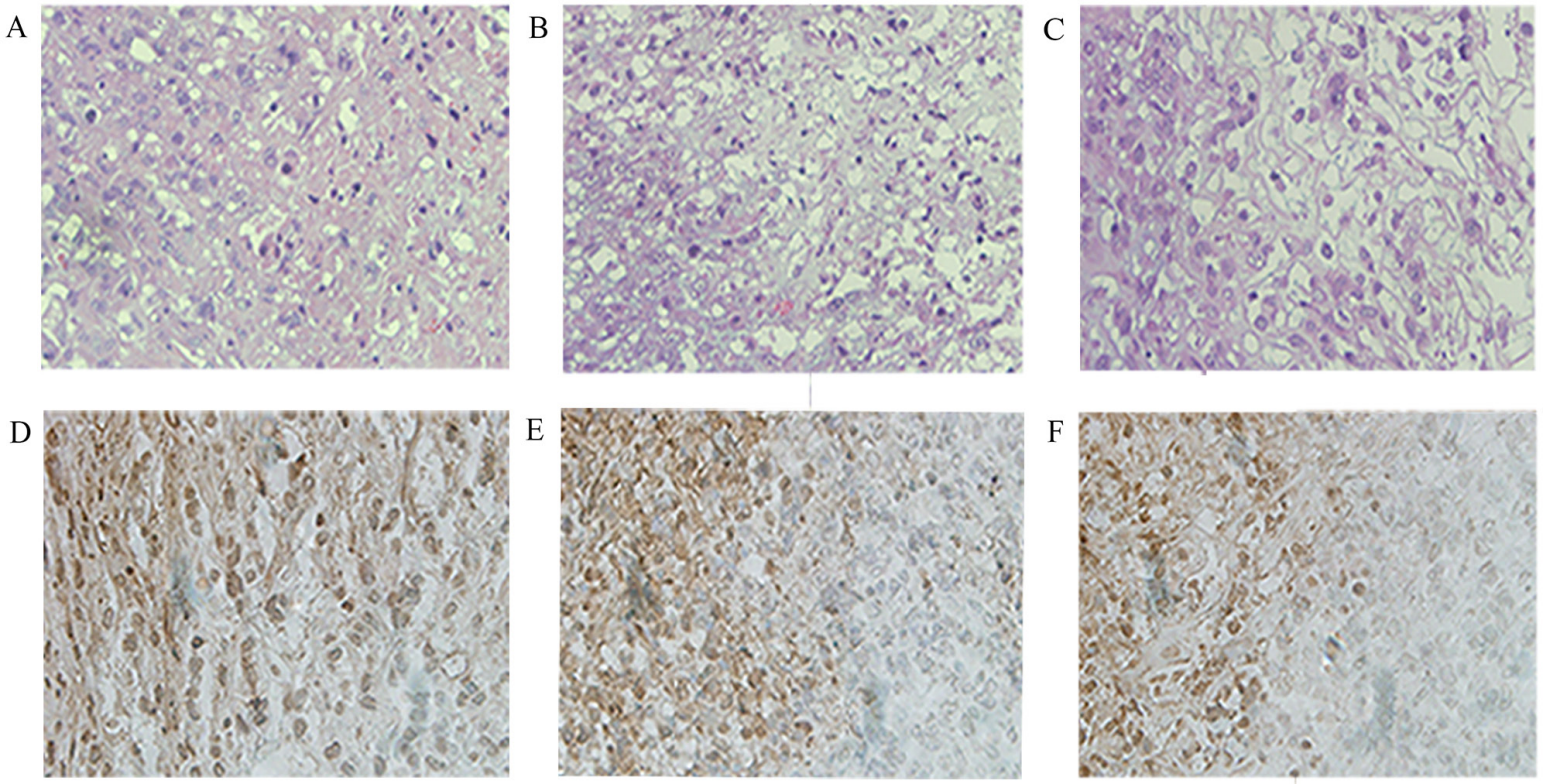
Histopathologic analysis in Group B at different times after HIFU irradiation Necrotic areas were identified by use of hematoxylin and eosin (H&E) staining under a high-power field (×400) (**A, B, and C**). Apoptotic cells were quantified using the TUNEL assay (×400) (**D, E, and F**). There were significant differences of apoptosis and necrotic areas of the tumor between different times after HIFU irradiation.

Within 24 hours of HIFU irradiation, the interior of the xenografts showed no significant changes on B-mode US examination, still exhibiting low or very low echo. Some xenografts had small patches of slightly high echo inside the tumors (Figure 2B). Color Doppler flow imaging revealed no significant detectable blood flow within the xenografts, but the periphery of the xenografts showed visible dot-like flow signals (Figure 2F). CEUS revealed contrast agent perfusion at the periphery of the tumors, as well as irregular un-perfused areas within the tumors (Figure 3B). The histological result suggested that cells within the ablation lesions showed apoptosis and necrosis 24 hours after HIFU irradiation. There were no clear boundaries between the injured areas and the surrounding normal tumor cells. Because of the expansion of microvascular congestion within the xenografts at the early stage after irradiation, multiple small vessel ruptures occurred within the ablation lesions (Figure 4A). As shown in the Figure 4D, 4E, and 4F, some cells within the ablation lesions showed apoptosis 24 hours after HIFU irradiation. Apoptotic nuclei showed clear brown staining, suggesting the formation of DNA fragments (Figure 4D). Clear chromatin margination was observed in the nuclei, and formation of apoptotic bodies in the cells was also observed. At 48 hours after HIFU irradiation, B-mode US examinations demonstrated low or very low echo within the tumors (Figure 2C), with no significant changes compared with the pre-treatment conditions. Color Doppler flow imaging did not reveal significant blood flow within the tumors, and the dot-like blood flow signals in the xenograft periphery were decreased. (Figure 2G). The CEUS results were like those of the 24-hours group, with irregular un-perfused areas within the tumors and contrast agent perfusion in the tumor periphery (Figure 3C). At 48 hours after HIFU irradiation, ablation lesions gradually showed clear boundaries. The distribution of small blood vessels within the ablation lesions was reduced, and cellular structures were damaged and became fuzzy. Tissues in the target areas showed large patches of necrosis and apoptosis (Figure 4B). At 48 hours after HIFU irradiation, the ablation lesions still contained many apoptotic and necrotic cells (Figure 4E).

At 2 weeks after HIFU irradiation, B-mode US demonstrated that the echo within the tumor was not significantly increased (Figure 2D). Color Doppler flow imaging showed that the blood flow signals in the periphery of xenografts were increased (Figure 2H). CEUS revealed un-perfused areas within the tumors that enlarged with time after HIFU irradiation. The peripheral contrast agent perfusion area was increased compared with the 24-hours and 48-hours groups (Figure 3D). At 2 weeks after HIFU irradiation, the tissues in the ablation zone showed homogeneous cellular structures. The tissues exhibited significant organization and fibrosis. Tissue dissolution and absorption were found between necrotic tissues, leading to the formation of small cavities (Figure 4C). At 2 weeks after HIFU irradiation, the number of apoptotic cells decreased in the ablation lesions. Only a small number of necrotic cells remained, with nuclear condensation and dark brown-stained nuclei (Figure 4F).

DCE-US was performed before HIFU irradiation and at 24 hours, 48 hours, and 2 weeks after irradiation in the sub-ablation group. Four DCE-US perfusion parameters were computed as shown in Table 3. Correlation between tumor size and US perfusion parameters was analyzed. DCE-US perfusion parameters, including peak intensity (PI) (58.3 ± 7.1 vs. 20.4 ± 5.2), area under the curve (AUC) (13.2 ± 3.2 vs. 11.3 ± 4.1), had decreased 1 day after irradiation. In addition, TP (12532.7 ± 34.9 vs. 15721.4 ± 54.2) and sharpness (0.24 ± 0.1 vs. 0.54±0.2) increased at 24 hours after irradiation. However, PI and AUC increased at 48 hours and 2 weeks after irradiation compared with 24 hours after irradiation. Time to peak (TP) decreased compared with those of the 24-hours group.

**Table 3:** US perfusion parameters before and after sub-threshold HIFU irradiation in Group B

	Before HIFU	24 hours after HIFU irradiation	48 hours after HIFU irradiation	2 weeks after HIFU irradiation
PI	58.3 ± 7.1	20.4 ± 5.2*	27.9 ± 4.9*	32.1 ± 6.4*
TP	12532.7 ± 34.9	15721.4 ± 54.2*	13356.1 ± 75.5	13734.8 ± 45.9
Sharpness	0.24 ± 0.1	0.54 ± 0.2*	0.78 ± 0.3*	0.61 ± 0.2*
AUC	13.2 ± 3.2	11.3 ±4.1*	19.5 ± 3.4*	22.2 ± 5.9*

Note: Values are expressed as mean ± SD.

PI: Peak intensity. TP: Time to peak. AUC: Area under the curve.

*P <0.05 compared to parameters before HIFU irradiation.

**Figure 5:**
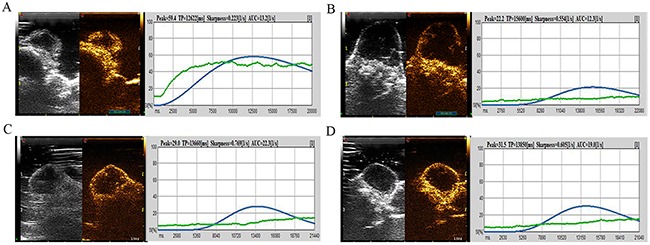
CEUS image and DCE-US time-intensity curves of one mouse in Group B at different times before and after irradiation **(A)** The CEUS image and DCE-US perfusion parameters before HIFU irradiation. **(B)** The CEUS image and the intensity of ultrasonic image changing over time at 24 hours after HIFU irradiation. **(C)** Perfusion parameters at 48 hours after HIFU irradiation. **(D)** Time-intensity curve at 2 weeks after HIFU irradiation.

**Figure 6:**
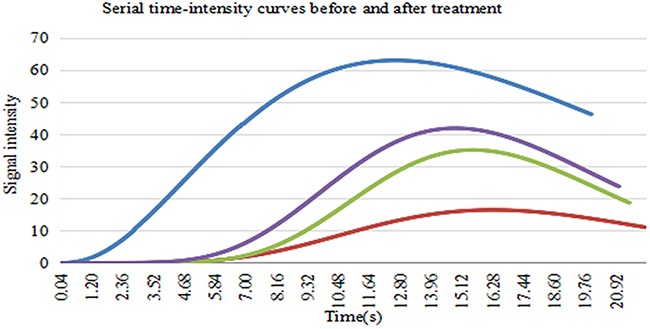
The DCE-US time-intensity curves of one mouse in Group B at different times before and after irradiation Blue line shows the DCE-US time-intensity curve before HIFU irradiation. Red line shows the intensity of the ultrasonic image changing over time at 24 hours after HIFU irradiation. Green line shows the time-intensity curve at 48 hours after HIFU irradiation. Purple line shows time-intensity curve at 2 weeks after HIFU irradiation.

CEUS images and DCE-US time-intensity curves of one mouse in Group B at different times, before and after irradiation, are shown (Figure 5). In Figure 6, the blue line with the highest PI and least TP shows the DCE-US time-intensity curve before HIFU irradiation. The red line with the lowest PI and longest TP reflects the intensity of the ultrasonic image changing over time at 24 hours after HIFU irradiation. The green line and the purple line show the time-intensity curve at 48 hour and 2 weeks after HIFU irradiation, respectively, which are smoother than the blue line.

## DISCUSSION

As a noninvasive tumor treatment technology, HIFU cannot be evaluated for efficacy without imaging methods. Accurate and objective determination of post-treatment changes in lesions *in vivo* is the key to guaranteeing its efficacy [15, 16]. The advantages of US monitoring are that it is conducted in real time and it is compatible with HIFU transducers. CEUS can also be used to monitor HIFU irradiation and to evaluate lesions after HIFU ablation [17]. Because changes of the acoustic attenuation coefficient and the backscatter coefficient that occur after HIFU irradiation in tissues are not significant compared with the surrounding normal tissue, the boundaries of HIFU ablation lesions are often not clearly displayed on B-mode images [18, 19]. In this study, an HIFU acoustic energy dose at the sub-threshold ablation temperature was used, and the tissue temperature was not raised above 60° C. No significant ultrasound imaging changes were observed after tissue injury, and xenografts before and after irradiation showed low to very low echo in the B-mode US images.

For blood-rich lesions, color Doppler flow imaging can be used to determine tissue survival by the reduction or elimination of the blood-flow distribution in the ablation lesions after HIFU irradiation [20, 21], which is more difficult to determine for lesions with less blood flow. In this study, because of the color Doppler flow imaging resolution and the limitation of lower blood flow velocity within small tumors, color Doppler flow imaging could only display the blood-flow signals of large, nourished blood vessels in the tumor periphery. The interior of the xenografts showed no significant blood flow before or after irradiation; therefore, we could not evaluate the changes in the blood-flow distribution within xenografts after HIFU irradiation.

CEUS can be used to observe changes in blood perfusion in the ablation areas after HIFU irradiation. When high acoustic energy doses of HIFU radiation are used to produce coagulation necrosis in tumor tissues, vascular structures inside the tissues can be damaged [22]. This damage can result in un-perfused areas with hypoecho. Therefore, CEUS imaging can clearly reveal the morphology and extent of areas with impaired perfusion in the HIFU ablation lesion [23, 24] and assess the residual tumor tissue. Because we used HIFU at the critical temperature(55°-60°C) for tumor ablation to irradiate pancreatic cancer xenografts, the injury effects on small vessels were not obvious. The pathological results at the early stages after irradiation showed that the vasculature inside the xenografts was not completely destroyed, and local distributions of small blood vessel congestion in the ablation lesions were observed. Therefore, at the early stages after irradiation, CEUS showed patchy non-perfusion zones, and the periphery of the tumors still showed string-like and floc-like contrast-agent perfusion. At 2 weeks after HIFU irradiation, tissues in the ablation region showed organization and fibrosis, the internal vascular structure gradually disappeared, the ablation lesions gradually showed clear boundaries, and CEUS scans displayed the extent and region of HIFU ablation lesions more clearly.

At the early phase after HIFU irradiation at the critical ablation temperature (55°C-60°C), CEUS examinations showed heterogeneous partial tumor perfusion caused by incomplete blocking of the small blood vessels by congestion in the target area. The biological effects of HIFU irradiation at the critical temperature(55°C-60°C) for ablation are not sufficient to cause instant tissue necrosis. Instead, HIFU irradiation inhibits tumor growth by inducing apoptosis. Therefore, an inaccurate evaluation of the early efficacy of HIFU irradiation is possible with the use of contrast-enhanced imaging in the ablation areas, resulting in excessive radiation. DCE-US might serve as a surrogate marker for predicting the treatment response, which was useful for evaluating tumor vascular changes with DCE-US [21, 23]. Four DCE-US parameters, PI, AUC, TP, and sharpness, were significantly correlated with the treatment response after a few days or weeks of irradiation. Kim et al [14] demonstrated that US perfusion parameters, including PI and AUC, had already decreased significantly after the first day of irradiation (*P* <0.05) in the combined gemcitabine and low-power HIFU irradiation group. Gemcitabine was administered by intraperitoneal injection at 100 mg/kg once a day every 3 days for a total of 4 injections. Their study, allowed earlier modification and establishment of a treatment regimen because DCE-US allowed assessment of the treatment response much earlier than by monitoring tumor volume changes that occurred in the later course of treatment.

As shown in this study, HIFU (Group C, 65°C-80° C) and sub-threshold HIFU (Group B, 55°C-60° C) inhibits tumor growth. Three tumors in Group C disappeared, with seven mice skin burned. In Group B, all tumors showed inhibited growth, with no mice skin burned. The US perfusion parameters, including PI and AUC, had decreased significantly 24 hours after irradiation (*P* <0.05) in Group B. TP and sharpness had increased significantly 24 hours after irradiation because the tumor was ablated and the volume of the tumor was reduced. At the late phases of irradiation, PI and AUC increased and TP decreased at 48 hours and 2 weeks after irradiation compared with 24 hours because the tumor had grown larger and microvascular increased. As shown in Figure 6, the red line with the lowest PI and longest TP at 24 hours after HIFU irradiation might reflect the best efficiency of sub-threshold HIFU radiation. Because of the tumor growth and blood vessel density inside the tumor increased, green line and the purple line are both above the red line. These results suggest that the US perfusion parameters are useful for predicting the therapeutic response after irradiation.

Our study had some limitations: We did not have the tools for measuring cavitation and temperature in our HIFU setting. Monitoring cavitation and temperature is important in understanding the pathophysiology and further improving the efficacy of HIFU irradiation. In our study, DCE-US was performed before and 24 hours, 48 hours, and 2 weeks after irradiation to predict the treatment response. Three tumors disappeared and the tumor volumes in the other seven mice apparently decreased in Group C. Because the tumors were too small to use DCE-US to evaluate the treatment response at 2 weeks after irradiation, we did not use DCE-US to follow the treatment of Group C as we did in Group B.

## MATERIALS AND METHODS

### Establishment of a subcutaneous xenograft model of pancreatic cancer in nude mice

The human pancreatic cancer cell line PaTu 8988t was a gift from Professor Xingpeng Wang at the Shanghai First People's Hospital. This cell line was derived from human pancreatic cancer liver metastases and grows as adherent cells. PaTu 8988t cells were cultured in RPMI-1640 medium (containing 10% fetal bovine serum, 100 U/mL penicillin, and 100 U/mL streptomycin), maintained in a 37 °C and 5% CO_2_ incubator, and passaged every other day.

Thirty female BALB/c-nu/nu nude mice (Shanghai Laboratory Animal Center, Chinese Academy of Science) aged 4 to 6 weeks, weighing 20 ± 2.0 g, were reared in specific pathogen free (SPF) clean rooms at the Shanghai Sixth People's Hospital Experimental Animal Center. All the procedures regarding animal maintenance and experiments are in strict accordance with the policy of the Institutional Animal Care and Use Committee (IACUC) of Shanghai Jiaotong University affiliated with Shanghai Sixth People's Hospital. The IACUC approved this study. The nude mice were anesthetized by intraperitoneal injection of sodium pentobarbital at 50 mg/kg, and all efforts were made to minimize suffering.

PaTu 8988t pancreatic cancer cells were diluted and re-suspended with RPMI-1640 medium and made into a single-cell suspension. The trypan blue exclusion method was used to count the number of living cells, ensuring a rate of living cells > 95%. After centrifugation, the supernatant was discarded, the cells were washed twice in PBS, and the cells were re-suspended in normal saline. To establish a tumor model, 1 × 10^7^ PaTu 8988t cells in 0.2 mL of cell suspension were subcutaneously injected into the right shoulders of the nude mice. The growth status of the nude mice and the tumor growth conditions were observed every 3 days. The tumor size was measured with a Vernier caliper. The longer diameter was recorded as L, the width diameter was recorded as W, and the tumor volume V was calculated according to the formula V = 1/2 (L × W^2^) [15].

### Laboratory instruments and equipment

#### HIFU irradiation equipment

The HY2900 HIFU therapy system (Wuxi Haiying Technology, Wuxi, China) was used in this study. A 3.5 MHz diagnostic transducer was positioned in the center of the therapeutic transducer to guide and monitor the HIFU irradiation.

The temperature-sensing needle, which is a thermocouple, was provided by the Jiangsu Wuxi Haiying Medical System Co., Ltd. The needle was 200 mm in length with an outer diameter of 1mm, and the temperature sensing point was at the tip of the needle.

#### US imaging equipment

A MyLab^™^ 90 color Doppler US instrument with X-Contrast Tuned Imaging (X-CnTI) technology was used in this study. The US probe was a LA522 linear probe, and the contrast agent used was SonoVue™ from Bracco (Milan, Italy).

### Experimental methods

Thirty tumor-bearing nude mice were divided into 3 groups. In Group A (control group), 10 tumor-bearing nude mice received sham irradiation. In Group B, 10 mice received the moderate acoustic energy dose (sub-ablation) HIFU irradiation. In Group C, 10 mice received the high acoustic energy dose HIFU irradiation. All animals were dosed when the tumor volume reached approximately 500 mm^3^. Before all imaging experiments, a simple, small-animal protection mold was built of sound-absorbing rubber sheets. The tumors of the nude mice could pass through an approximately 1.2-cm hole in the mold. The contact surfaces of the upper and lower ends were treated with a polyacrylamide gel and an ultrasonic couplant

The HIFU irradiation regimen used a single-point, single-time irradiation mode. T ON (the irradiation time at each single point) was set to 500 ms and T OFF (the irradiation interval time) was set to 2000 ms. The output acoustic power (P) of the Group A was set to 0 W. The P was set to 47.92 W in Group B and 119.8 W in Group C. The interval between two points was 1 mm, and the spatially averaged intensity was 1134W/cm^2^ in Group B and 2835 W/cm^2^ in Group C. The tip of the temperature-sensing needle was inserted approximately 4 mm into the tumor, to monitor the tumor temperature during irradiation and to ensure that the maximum intratumoral temperature was 55°-60° C in Group B and 65°-80° C in Group C. The whole tumor was irradiation.

After sub-threshold HIFU irradiation in Group B, B-mode US, color Doppler imaging and power Doppler US examinations were conducted. The examination time was chosen to be before HIFU irradiation and at 24 hours, 48 hours, and 2 weeks after irradiation. Examination variables such as the probe frequency, gain, focus points, focus range, and depth were adjusted to obtain the best images. Conventional B-mode US was used to observe the echo types and echo distribution within the tumor before and after irradiation, and color or power Doppler US examinations were used to observe blood flow within and around the pancreatic cancer xenografts.

### DCE-US study and image analysis

DCE-US was scheduled before HIFU irradiation and at 24 hours, 48 hours, and 2 weeks after irradiation. Baseline and DCE-US examinations before and after the irradiation were performed by an ultrasonographer using a MyLab™ 90 ultrasound unit (Italy) with a 12-MHz LA522 linear probe. The parameters were as follows: PEN-M, D29, PRC13/3/2, PRS6, PST1, C10, DP40KPa, RES-M, CnTI PRC9/3/2, PRS0, PST0, XV off, 13 Hz, and a depth of 25 mm. First, a morphological study was performed in B mode US to find the tumor and measure its maximum dimensions. The tumor was then imaged by use of the maximum transverse planes, which were measured accordingly. An indwelling intravenous infusion needle was inserted into the tail veins of the nude mice. The US contrast agent was mixed by shaking before use. Subsequently, 0.2 mL of US contrast agent microbubbles (SonoVue, Bracco; Milan, Italy) were aspirated with a 1-mL syringe and injected by use of the bolus injection method via the tail vein, followed by flushing with 1 mL of normal saline. The maximum cross-sectional plane of the xenograft was selected as the imaging observation plane, a timer was started, and a video recording was started at the time of contrast agent injection. To obtain DCE-US data, all ultrasonographic examinations were conducted with the same instrument settings. All injections of contrast agent were done by the same experimenter. All recorded data were analyzed by dedicated QontraXt Trial V2.0 software (Esaote; Genoa, Italy). A region of interest (ROI) was manually drawn along the tumor margin in a selected frame, and then auto-positioned on all the images of the study. If changes in tumor position occurred because of respiratory motion during the examination, the ROI was adjusted for those frames, and the software interpolated the ROI positions between the two frames of different ROIs and automatically tracked the later ROI. From the time-intensity curves of the ROIs, which were generated by analyzing the 3-minute raw data of the DCE-US imaging, the following perfusion parameters were obtained: PI, TP, sharpness, and AUC.

### Pathology

Before HIFU irradiation, and 24 hours, 48 hours, and 2 weeks after HIFU irradiation, after euthanizing the mice, representative specimens were obtained from each group. For each tumor tissue, hematoxylin and eosin (H&E) and terminal deoxynucleotidyl transferase-modified, dUTP nick-end labeling (TUNEL) staining (Millipore; Bedford, MA, USA) were performed to evaluate the necrotic fraction and apoptosis of the tumor, respectively. Necrotic areas were identified by use of H&E staining under a high-power field (×400) and apoptotic cells were quantified by use of the TUNEL assay under a high-power field (×400) also.

### Statistics and analysis

Differences in tumor size and DCE-US perfusion parameters before and after irradiation were assessed using the paired *t*-test. A *P* value less than 0.05 indicated a statistically significant difference. Statistical analyses were conducted by application of an SPSS software package (Version 14.0, SPSS).
